# Control of Amygdala Circuits by 5-HT Neurons via 5-HT and Glutamate Cotransmission

**DOI:** 10.1523/JNEUROSCI.2238-16.2016

**Published:** 2017-02-15

**Authors:** Ayesha Sengupta, Marco Bocchio, David M. Bannerman, Trevor Sharp, Marco Capogna

**Affiliations:** ^1^Department of Pharmacology, University of Oxford, Oxford, OX1 3QT United Kingdom,; ^2^Medical Research Council Brain Network Dynamics Unit, Department of Pharmacology, University of Oxford, Oxford, OX1 3TH United Kingdom,; ^3^Department of Experimental Psychology, University of Oxford, Oxford, OX1 3UD United Kingdom,; ^4^Department of Biomedicine, University of Aarhus, 8000 Aarhus C, Denmark, and; ^5^The Danish Research Institute of Translational Neuroscience (DANDRITE), Nordic EMBL Partnership for Molecular Medicine, Department of Biomedicine, University of Aarhus, 8000 Aarhus C, Denmark

**Keywords:** amygdala, electrophysiology, interneuron, optogenetics, serotonin, synaptic transmission

## Abstract

The serotonin (5-HT) system and the amygdala are key regulators of emotional behavior. Several lines of evidence suggest that 5-HT transmission in the amygdala is implicated in the susceptibility and drug treatment of mood disorders. Therefore, elucidating the physiological mechanisms through which midbrain 5-HT neurons modulate amygdala circuits could be pivotal in understanding emotional regulation in health and disease. To shed light on these mechanisms, we performed patch-clamp recordings from basal amygdala (BA) neurons in brain slices from mice with channelrhodopsin genetically targeted to 5-HT neurons. Optical stimulation of 5-HT terminals at low frequencies (≤1 Hz) evoked a short-latency excitation of BA interneurons (INs) that was depressed at higher frequencies. Pharmacological analysis revealed that this effect was mediated by glutamate and not 5-HT because it was abolished by ionotropic glutamate receptor antagonists. Optical stimulation of 5-HT terminals at higher frequencies (10–20 Hz) evoked both slow excitation and slow inhibition of INs. These effects were mediated by 5-HT because they were blocked by antagonists of 5-HT_2A_ and 5-HT_1A_ receptors, respectively. These fast glutamate- and slow 5-HT-mediated responses often coexisted in the same neuron. Interestingly, fast-spiking and non-fast-spiking INs displayed differential modulation by glutamate and 5-HT. Furthermore, optical stimulation of 5-HT terminals did not evoke glutamate release onto BA principal neurons, but inhibited these cells directly via activation of 5-HT_1A_ receptors and indirectly via enhanced GABA release. Collectively, these findings suggest that 5-HT neurons exert a frequency-dependent, cell-type-specific control over BA circuitry via 5-HT and glutamate co-release to inhibit the BA output.

**SIGNIFICANCE STATEMENT** The modulation of the amygdala by serotonin (5-HT) is important for emotional regulation and is implicated in the pathogenesis and treatment of affective disorders. Therefore, it is essential to determine the physiological mechanisms through which 5-HT neurons in the dorsal raphe nuclei modulate amygdala circuits. Here, we combined optogenetic, electrophysiological, and pharmacological approaches to study the effects of activation of 5-HT axons in the basal nucleus of the amygdala (BA). We found that 5-HT neurons co-release 5-HT and glutamate onto BA neurons in a cell-type-specific and frequency-dependent manner. Therefore, we suggest that theories on the contribution of 5-HT neurons to amygdala function should be revised to incorporate the concept of 5-HT/glutamate cotransmission.

## Introduction

The neurotransmitter serotonin (5-hydroxytryptamine; 5-HT) regulates a vast array of brain functions. Among these, 5-HT is strongly implicated in emotional regulation and in the etiology and drug treatment of mood and anxiety disorders (Nemeroff and Owens, 2002). Presumably, these behavioral effects arise from the 5-HT modulation of multiple neuronal circuits, but 5-HT transmission in the amygdala is likely to play a critical role. The amygdala is a brain region that comprises multiple nuclei that collectively encode many aspects of emotionality including fear learning and memory (Duvarci and Pare, 2014; Janak and Tye, 2015). Across species, the amygdala is heavily innervated by 5-HT neurons arising from the dorsal raphe nucleus (DRN) in the midbrain (Jacobs et al., 1978; Steinbusch, 1981; Asan et al., 2013). Moreover, selective pharmacological and optogenetic manipulations of 5-HT in the amygdala are reported to have striking effects on fear and anxiety responses in experimental animals (Christianson et al., 2010; Johnson et al., 2015; Baratta et al., 2016).

The interaction between 5-HT and the amygdala is complex and takes the form of synaptic and nonsynaptic 5-HT terminals that target diverse excitatory and inhibitory 5-HT receptor subtypes distributed across a microcircuitry comprised of many neuron populations (for review, see Asan et al., 2013; Bocchio et al., 2016b). In the basolateral amygdala (BLA), further subdivided into basal amygdala (BA) and lateral amygdala (LA), immunohistochemical and *in situ* hybridization studies (Aznar et al., 2003; McDonald and Mascagni, 2007) report the presence of both excitatory (5-HT_2A_) and inhibitory (5-HT_1A_) 5-HT receptors on glutamatergic principal neurons (PNs), which comprise ∼80% of the neurons of the BLA. Similar studies report that these receptors, as well as excitatory ionotropic (5-HT_3A_) 5-HT receptors, are also distributed across different groups of GABAergic interneurons (INs) in the BLA (Aznar et al., 2003; Mascagni and McDonald, 2007; McDonald and Mascagni, 2007; Bonn et al., 2013).

These receptor localization studies are supported by investigations of 5-HT receptor function in the BLA. *In vitro* electrophysiological studies have found that PNs respond to exogenous 5-HT with 5-HT_2C_-receptor-mediated depolarization in the LA (Yamamoto et al., 2014), but not in the BA (Rainnie, 1999; Bocchio et al., 2015). Furthermore, 5-HT was found to depolarize BA INs, mainly via 5-HT_2A_ receptors, leading to enhanced GABA release onto PNs (Rainnie, 1999; Jiang et al., 2009; Bocchio et al., 2015).

Despite these advances, it remains crucial to understand how BA neurons respond to physiologically released 5-HT. Virtually all that is known about the action of 5-HT on BA neurons is based on the prolonged application of exogenous 5-HT and 5-HT ligands (Eidelberg et al., 1967; Rainnie, 1999; Stutzmann and LeDoux, 1999; Jiang et al., 2009; Bocchio et al., 2015). A pitfall of this approach was highlighted recently in a study finding that the response of BA neurons to acetylcholine released physiologically by targeted optogenetic stimulation did not match the canonical response of BA neurons to locally applied acetylcholine (Unal et al., 2015). Differences between such pharmacological and physiological approaches could arise from a mismatch between pharmacological and physiological concentrations of 5-HT, nonphysiological activation of extrasynaptic receptors, and the potential role of physiologically released cotransmitters. In the case of 5-HT, cotransmitters include a number of candidate amino acids and neuropeptides (Fernandez et al., 2016).

The current study adopted a more physiological approach to investigate the effect of 5-HT on BA neuron subtypes as a follow-up to our recent pharmacological 5-HT study (Bocchio et al., 2015). We stimulated 5-HT axons in the BA by the light activation of channelrhodopsin (ChR2), which was expressed selectively in DRN 5-HT neurons. Our results identify novel, cell-type-dependent mechanisms of control of amygdala microcircuits by DRN 5-HT neurons.

## Materials and Methods

### 

#### 

##### Animals.

SERT-Cre^+/−^ mice (MMRRC, B6.Cg-Tg(Slc6a4-cre)Et33Gsat, stock number 031028-UCD) of either sex were housed with their littermates with *ad libitum* access to food and water at a constant temperature and humidity on a 12/12 h light/dark cycle (lights on at 7:00 A.M.). Experiments were performed in compliance with the Animals (Scientific Procedures) Act of 1986 (UK) and approved by local ethical review at the University of Oxford.

##### Viral transfection.

To transfect 5-HT neurons selectively with ChR2, a Cre-inducible recombinant viral vector (AAV-EF1a-DIO-hChR2(E123T/T159C)-EYFP; UNC Vector Core) carrying a floxed ultrafast (E123T/T159C) ChR2 fused with yellow fluorescent protein (YFP) was stereotaxically injected (1 μl at 100 nL/min) into the DRN (coordinates in millimeters according to bregma and the brain surface; anterior–posterior −4.1; dorsoventral −2.5, −2.2, −1.9) of SERT-Cre mice at postnatal day 30 (P30)–P75 anesthetized using 1–2% isoflurane in oxygen (2 L/min). On recovery from surgery, mice were administered 0.3 mg/kg buprenorphine subcutaneously for postoperative analgesia.

##### Electrophysiological recordings.

Electrophysiological recordings were performed 4 weeks after viral transfection. Mice were deeply anesthetized (4% isoflurane) and brains were rapidly extracted and immersed in an ice-cold cutting solution composed of the following (in mm): 0.5 CaCl_2_, 10 glucose, 2.5 KCl, 7 MgCl_2_, 85 NaCl, 25 NaHCO_3_, 1.25 NaH_2_PO_4_, and 65 sucrose (all from Sigma-Aldrich) saturated with 95% O_2_, 5% CO_2_, at pH 7.3. Coronal slices (325 μm) were cut at 4°C at the level of the BLA or DRN using a microtome (Microm HM 650 V; Thermo Fisher Scientific). Slices were then incubated at 36°C for 10 min, after which heating was switched off to allow the slices to reequilibrate to room temperature (18–22°C, ∼1 h). During this period, slices were perfused at a rate of ∼5 ml/min with artificial CSF (ACSF) composed of the following (in mm): 2 CaCl_2_, 10 glucose, 3.5 KCl, 2 MgCl_2_, 130 NaCl, 24 NaHCO_3_, and 1.25 NaH_2_PO_4_; saturated with 95% O_2_, 5% CO_2_, at a pH 7.3. Slices were then transferred to a submerged recording chamber mounted on the stage of an upright microscope (Axioskop 2FS; Zeiss), secured under a nylon mesh, and perfused continuously with oxygenated ACSF at a rate of ∼5 ml/min at 34 ± 1°C.

Neurons were visualized using a 60× immersion objective (Olympus) coupled with infrared and differential interference contrast optics linked to a video camera (CCD Camera Orca R2; Hamamatsu). Somatic whole-cell patch-clamp recordings were made from visually identified BLA or DRN cells. Patch recording electrodes were pulled (DMZ puller; Zeitz Instruments) from borosilicate glass capillaries (GC120F, 1.2 mm outer diameter; Clarke Electromedical Instruments) and filled with a filtered solution consisting of the following (in mm): 140 K-gluconate, 4 KCl, 4 ATP-Mg, 0.3 GTP-Na_2_, 10 Na_2_-phosphocreatine, 10 HEPES, along with 0.5% w/v biocytin, osmolarity 270–280 mOsmol/L without biocytin, pH 7.3, adjusted with KOH. The patch electrode resistance was 4–6 MΩ. Electrophysiological signals were amplified (EPC9/2 amplifier; HEKA Electronik), acquired using Patchmaster software (HEKA Electronik), low-pass filtered at 2.9 kHz, and digitized at 5 or 10 kHz. Recordings were accepted only when the initial seal resistance was >2 GΩ and the series resistance (22.8 ± 0.9 MΩ, range: 11–26 MΩ) did not change by >20% throughout the recording. No correction was made for the junction potential between the pipette and the ACSF (14 mV).

Patch-clamp recordings were targeted at the BA subdivision of the BLA because the BA had the densest ChR2/YFP+ projections from the DRN (see below). During optical stimulation protocols in voltage-clamp mode, PNs were held at −70 mV to test for the presence of fast and slow EPSCs and then at −50 mV to monitor both slow 5-HT-evoked IPSCs and spontaneous IPSCs (sIPSCs). The INs were always held at −70 mV because this allowed study of the kinetics of 5-HT-mediated slow IPSCs and EPSCs at the same time. Importantly, we verified in a subset of cells (*n* = 8/8) that neurons displaying a significant 5-HT_1A_-mediated slow IPSCs at −50 mV (see below for criteria of significance) also displayed a significant slow IPSC at −70 mV. This suggests that the holding potential is unlikely to have affected the relative distribution of response types in INs (see Fig. 4*A*).

Optical stimulation of ChR2-expressing 5-HT afferents in the BLA was performed using an optoLED system (Cairn Research) consisting of a 3.5 W LED mounted on a Zeiss Axioskop 2 FS microscope, which delivered pulses of light (470 nm) of 3–5 ms duration and 8 mW/mm^2^ intensity. The spot size corresponded to the area of slice visualized with a 60× objective. Single light pulses were delivered at 20 s intervals and repeated at least 10 times. Optical stimulation trains consisted of 5 or 30 s trains of light pulses delivered at 1, 10, or 20 Hz. To avoid adaptation of responses due to receptor desensitization, 10 min intervals were allowed between subsequent 30 s trains.

##### Data analysis.

Analyses of synaptic currents and intrinsic membrane properties were performed using IGOR Pro (Wavemetrics) and MATLAB (The MathWorks). The rheobase (in picoamperes) was determined as the 50 ms current injection able to generate a spike in 50% of the cases of 10 trials. Instantaneous firing rate (in Hertz) was defined as the number of action potentials evoked during a 1-s-long depolarizing current pulse of twice the amplitude of the rheobase current. Adaptation index (range: 0–1) was defined as the ratio between the first and last interspike interval (ISI, in milliseconds) elicited by the same current pulse used to measure instantaneous firing rate. The coefficient of variation (CV) of the ISI was calculated from the instantaneous firing rate and was the ratio of the ISI SD and ISI mean.

BA PNs were distinguished from INs according to the following electrophysiological parameters, consistent with previous reports (Sosulina et al., 2006; Bocchio et al., 2015): (1) smaller amplitude of fast after-hyperpolarization (AHP) and prominent medium AHP from the second spike in an instantaneous firing rate protocol; (2) adapting ISIs; (3) instantaneous firing rate <20 Hz; (4) lower input resistance (*R*_in_, <150 MΩ); and (5) longer spike half-width (∼1 ms). INs were divided into fast-spiking and non-fast-spiking categories according to previous electrophysiological criteria (Rainnie, 1999; Ascoli et al., 2008), with minor adaptations. Specifically, fast-spiking INs had instantaneous firing rates of ≥50 Hz, an adaptation index of ≥0.4, a CV of the ISI of <0.8, and a spike half-width of 0.33 ± 0.01 ms, whereas non-fast-spiking neurons had instantaneous firing rates of <50 Hz, an adaptation index of <0.4, a CV ISI of ≥0.8, and a spike half-width of 0.51 ± 0.03 ms.

Postsynaptic currents were considered significant only if their amplitude was at least 2 SDs above or below the mean baseline current. Current amplitudes were calculated from the difference between the peak and mean baseline current. Spontaneous IPSCs were detected using TaroTools toolbox for Igor Pro (https://sites.google.com/site/tarotoolsregister). At the end of recordings, some slices were fixed overnight at 4°C in 4% paraformaldehyde (PFA) and 15% saturated picric acid in 0.1 m PB. After 24 h, slices were embedded in gelatin and resectioned (60 μm) with a VT-1000 vibrating microtome (Leica).

##### Immunohistochemistry.

Sections containing biocytin-filled neurons were stained with Cy3-conjugated streptavidin (1:1000–3000; Life Technologies) and visualized using an epifluorescence microscope (AxioImager M2; Zeiss). Confocal stacks of axonal and dendritic fields of labeled neurons were acquired using a laser-scanning confocal microscope (LSM 510; Zeiss).

For immunohistological analysis, mice were perfused transcardially with 0.1 m phosphate buffer, pH 7.3, followed by a fixative solution containing 4% PFA and 15% (v/v) saturated picric acid in 0.1 m phosphate buffer, pH 7.3. Brains were extracted, postfixed overnight at 4°C in the same fixative solution, and coronal sections (60 μm) were cut with a VT-1000 vibrating microtome (Leica). Sections were stained with the following antibodies: goat anti-5-HT (1:500 dilution; Immunostar, catalog #20079), which was visualized with a DyLight 594-conjugated secondary antibody (1:500 dilution; Abcam, catalog #ab96937); rabbit anti-5-HT (1:2500 dilution; kindly provided by Prof. Harry Steinbusch, Maastricht University, the Netherlands), which was visualized with a Cy3-conjugated secondary antibody (1:500 dilution; Jackson Immunoresearch, catalog #711-165-152); and guinea pig anti-VGLUT3 (1:250 dilution; kindly provided by Prof. Masahiko Watanabe, Hokkaido University Graduate School of Medicine, Sapporo, Japan), which was visualized with the same Cy3-conjugated secondary antibody. To strengthen the endogenous ChR2/YFP fluorescence (ChR2/YFP+), sections were stained with a chicken anti-GFP antibody (1:1000 dilution; Aves Laboratories) and Alexa Fluor 488-conjugated secondary antibody (Jackson Immunoresearch, catalog #703-545-155).

##### Quantification of 5-HT-immunoreactive ChR2/YFP-transfected neurons.

To quantify the percentage of ChR2/YFP+ neurons in the DRN that also expressed 5-HT, three evenly spaced coronal sections [rostrocaudal coordinates ∼−5 to −4.3 mm from bregma; from the Franklin and Watson (1997) atlas] were selected and a region of interest within the DRN was defined using a 5× 0.16 numerical aperture (NA) objective lens. A series of tiled stacked images (10 μm depth) were acquired using a 40× 1.3 NA oil-immersion objective and 1 μm steps. Counting was performed offline using StereoInvestigator (MBF Bioscience). A neuron was counted only if its immunopositive nucleus came into focus in the optical section. Nuclei already in focus at the top optical section were not counted (West, 1999). Data were exported to Excel (Microsoft) and pooled for further analysis.

##### Statistical analysis.

Data are presented as mean values ± SEM. For electrophysiological data, distributions passing the Shapiro–Wilk test for normality were compared using Student's *t* tests. Distributions that did not pass this test were compared using nonparametric tests (Mann–Whitney test, Wilcoxon matched-pairs signed-rank test). Comparisons between optical stimulation trains of different frequencies were performed with ANOVAs and Bonferroni *post hoc* tests. Correlations of response amplitudes were quantified using Pearson's correlation coefficient. Effects of optical stimulation on firing rate were analyzed with Dunnett's multiple-comparisons test. Statistical analysis was performed with SPSS or GraphPad Prism software. Differences were considered statistically significant at *p* < 0.05.

##### Drugs.

NBQX, APV, WAY 100635, SR 95531, and CGP54626 were from Tocris Biosciences. Kynurenic acid and MDL 100907 were from Sigma-Aldrich.

## Results

### Selective targeting of ChR2 expression to 5-HT neurons

**Figure 1.**
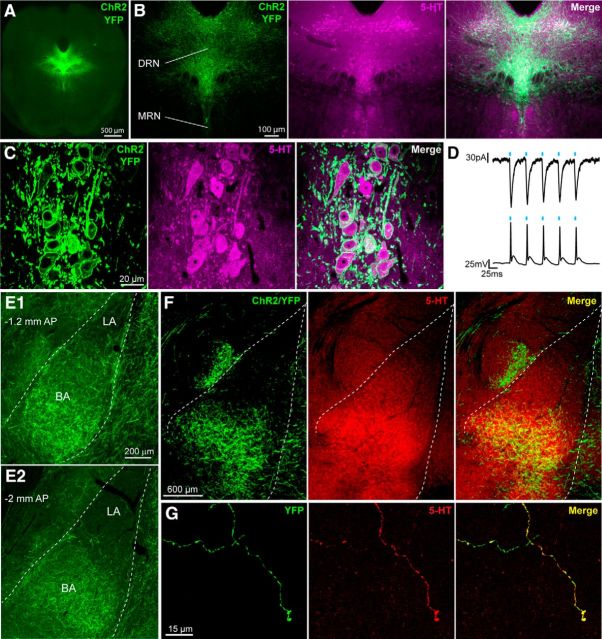
Expression of ChR2 in DRN 5-HT neurons. ***A***, Cre-dependent expression of ChR2/YFP in the DRN of SERT-Cre mice. ***B***, Overlap between ChR2/YFP and 5-HT expression in the DRN and lack of ChR2/YFP expression in the median raphe nucleus (MRN). ***C***, Maximum-intensity projection (*z*-stack: 4.2 μm, 0.21 μm optical sections) showing expression of ChR2/YFP in the membrane of 5-HT+ DRN neurons. ***D***, Light pulses (5 ms) delivered at 20 Hz evoke inward currents in voltage clamp (top) and action potentials in current clamp (bottom) in a DRN neuron expressing ChR2/YFP. ***E***, ChR2/YFP+ axons innervating the anterior (E1) and posterior BLA (E2); the BA shows a denser innervation than the LA. AP, Anteroposterior distance from bregma. ***F***, Overlap between 5-HT and ChR2/YFP expression in the BLA. ***G***, Representative image showing a 5-HT+ axon in the BA that also expresses ChR2/YFP.

To control 5-HT axons arising from the DRN and projecting to the BLA, a viral vector carrying a floxed ChR2 and tagged with YFP was stereotaxically injected into the DRN of SERT-Cre mice (Fig. 1*A*). Three weeks after injection of the viral vector, fluorescent microscopy analysis revealed an abundance of ChR2/YFP+ cell bodies within the DRN (Fig. 1*B*) that were 5-HT+ (Fig. 1*C*). Expression of ChR2/YFP+ was highly targeted in that 100% of ChR2/YFP+ cells (98.7 ± 22.5 ChR2+ cells counted per brain, *n* = 3 brains) were also 5-HT+. Two weeks after injection of the viral vector, the functionality of ChR2 expression was tested by whole-cell patch-clamp recordings from ChR2/YFP+ DRN neurons. Stimulation with pulses of light at 20 Hz reliably evoked inward currents in voltage-clamp mode, as well as action potentials in current-clamp mode (Fig. 1*D*), demonstrating selective light-gated control of DRN 5-HT neuron activity.

Four weeks after injection of the viral vector, brains of SERT-Cre mice displayed a widespread distribution of ChR2/YFP+ fibers, including an extensive innervation of the BLA (Fig. 1*E*,*F*). The expression pattern of ChR2/YFP in the BLA largely overlapped with that of 5-HT (Fig. 1*F*), with ChR2+/YFP+ axons being immunoreactive for 5-HT (Fig. 1*G*). Because the BA subdivision of the BLA displayed a particularly dense innervation of ChR2/YFP+ fibers compared with the LA (Fig. 1*E*,*F*), subsequent electrophysiological recordings were performed in the former region.

### Activation of 5-HT neurons evokes glutamate-mediated responses in BA INs

**Figure 2.**
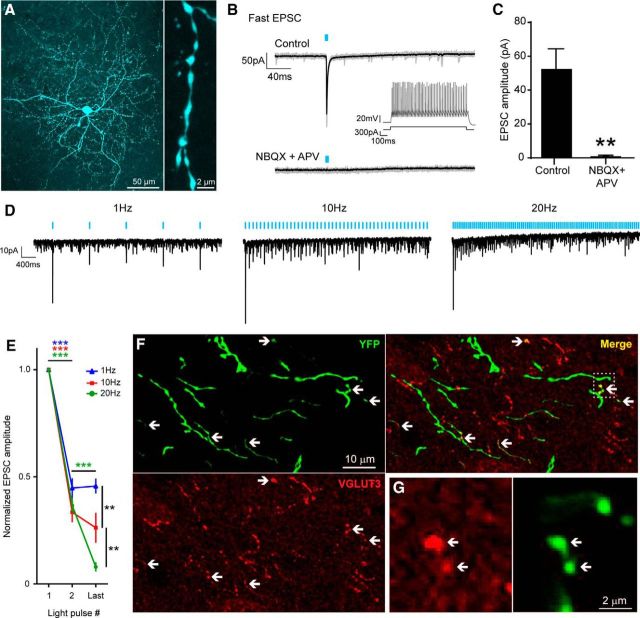
5-HT neurons release glutamate onto BA INs. ***A***, Representative biocytin-filled BA IN showing a dense local axon (left) and a spiny dendrite (right). ***B***, Example of a voltage-clamp (−70 mV) trace from a BA IN showing a fast EPSC evoked by a single light pulse (3 ms) activation of DRN axons (top); the fast EPSC is blocked by AMPA/kainate and the NMDA receptor antagonists NBQX (10 μm) and APV (50 μm; bottom). Black traces are the average of 10 individual traces, which are shown in gray. Inset, Firing pattern of a BA IN exhibiting the light-evoked fast EPSC. ***C***, Fast EPSC evoked in BA INs is blocked by NBQX and APV. (Wilcoxon matched-pairs signed-rank test, *p* = 0.0039, *n* = 9). ***D***, Example of a voltage-clamp (−70 mV) trace from a BA IN showing frequency-dependent depression of the fast glutamatergic EPSCs evoked by light trains (5 s) at 1 Hz (left), 10 Hz (middle), and 20 Hz (right). Traces shown are an average of five individual traces. ***E***, Stimulation-frequency-dependent depression of the second and last fast glutamatergic EPSC amplitudes normalized to the first EPSC amplitude for each cell. The depression of the last EPSC increases with higher stimulation frequencies (repeated-measures two-way ANOVA, main effect of light pulse no. *p* < 0.0001, main effect of stimulation frequency *p* = 0.0008; light pulse no. × stimulation frequency interaction *p* < 0.0001; Bonferroni's *post hoc* comparisons: at 1 Hz stimulation frequency, first vs second light pulse *p* < 0.0001, first vs last light pulse *p* < 0.0001; at 10 Hz stimulation frequency, first vs second light pulse *p* < 0.0001, first vs last light pulse *p* < 0.0001; at 20 Hz stimulation frequency, first vs second light pulse *p* < 0.0001, second vs last light pulse *p* < 0.0001, first vs last light pulse *p* < 0.0001; last light pulse, 20 Hz vs 10 Hz *p* = 0.0027, 10 Hz vs 1 Hz *p* = 0.0013, 20 Hz vs 1 Hz *p* < 0.0001; *n* = 7). ***F***, Maximum intensity projection (*z*-stack: 5.18 μm) showing sparse expression of VGLUT3 in 5-HT neuron axons (YFP+) in the BA. Arrows highlight YFP+/VGLUT3+ boutons. ***G***, Magnification of the area framed in ***F***. Single optical section (0.43 μm thickness) showing two YFP+ boutons immunopositive for VGLUT3. Data are expressed as means ± SEM. *p < 0.05; **p < 0.01; ***p < 0.0001.

Patch-clamp recordings were performed to investigate the mechanisms by which 5-HT axons derived from the DRN influence neurons within the BA microcircuit. BA INs were distinguished from PNs using electrophysiological criteria (see Materials and Methods) and the validity of these criteria was confirmed by *post hoc* histological analysis of cells (six of each type) filled with biocytin. In all cases, putative INs had the expected small, ovoid somata, spine-sparse dendrites, and axons that branched profusely within the BLA (Fig. 2*A*). In contrast, putative PNs had larger, pyramidal-shaped somata, thick spiny dendrites, and axons that often projected outside the BLA (see Fig. 5*A*). Because previous electrophysiological studies suggested that locally applied 5-HT depolarizes BA INs preferentially (Rainnie, 1999; Jiang et al., 2009; Bocchio et al., 2015), initial experiments investigated the effect of optical activation of 5-HT neurons on this cell population.

Single pulses of light (20 s intervals) elicited fast onset EPSCs, an effect observed in just over half (55%) of the INs tested (*n* = 59). Slow synaptic responses to single light pulses were not detected. Given the regular occurrence of this effect and the relative paucity of ionotropic 5-HT_3_ receptors in the BLA (Mascagni and McDonald, 2007), a 5-HT mechanism seemed unlikely. Indeed, these light-activated fast EPSCs were completely abolished by the combined application of NBQX (10 μm) and APV (50 μm), selective antagonists of AMPA/kainate and NMDA receptors, respectively (Wilcoxon matched-pairs signed-rank test, *p* = 0.0039, *n* = 9; Fig. 2*B*,*C*). In contrast, the EPSC amplitude was not altered by the 5-HT_3_ receptor antagonist MDL 72222 (20 μm, *n* = 2, data not shown). These results suggest that low-frequency stimulation of 5-HT terminals in the BA released glutamate preferentially over 5-HT to mediate ionotropic glutamate-receptor-mediated excitation of BA INs. Consistent with the release of glutamate from BA 5-HT axons, immunohistochemical experiments detected expression of the vesicular glutamate transporter VGLUT3 in ChR2/YFP+ axonal varicosities in the BA, although this colocalization was relatively sparse (Fig. 2*F*,*G*).

Next, the sensitivity of the glutamate-mediated fast EPSC to increasing stimulation frequencies (1, 10, and 20 Hz) was tested. Higher-frequency stimulation led to depression of fast EPSC amplitude (repeated measures two-way ANOVA, main effect of stimulation frequency *p* = 0.0008, *n* = 7 cells; Fig. 2*D*,*E*). These data suggest that the strength of glutamate-mediated excitation of INs by 5-HT neurons is greatest at lower firing rates of 5-HT neurons.

### Activation of 5-HT neurons also evokes 5-HT-mediated responses in BA INs

**Figure 3.**
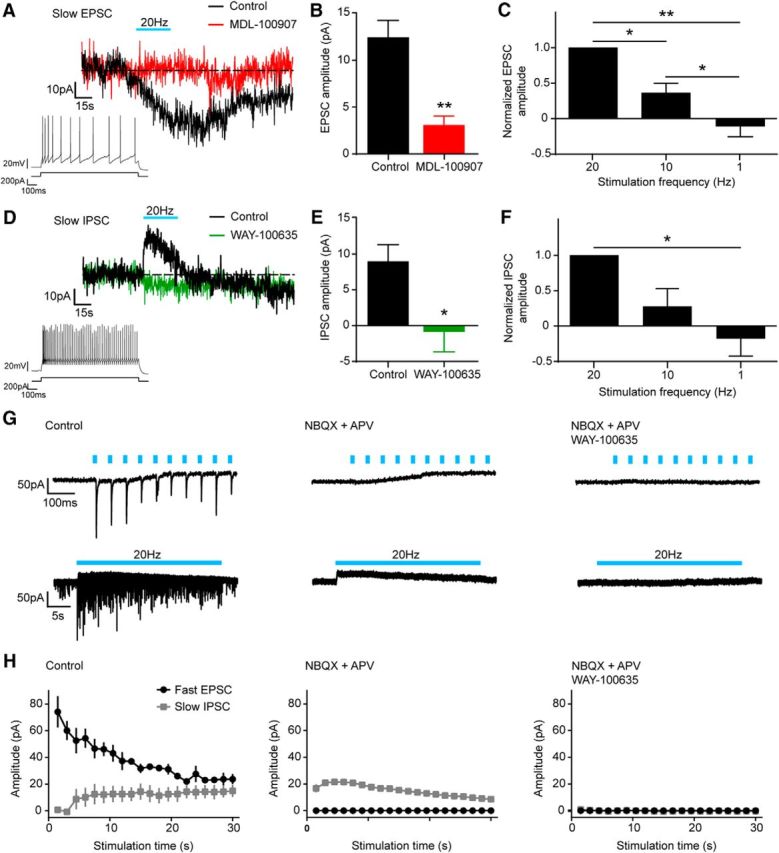
5-HT neurons modulate BA INs bidirectionally via 5-HT release. ***A***, Example of a voltage-clamp (−70 mV) trace from a BA IN showing a slow EPSC evoked by 30 s optical stimulation at 20 Hz of DRN axons. The slow EPSC is blocked by the 5-HT_2A_ receptor antagonist MDL 100907 (150 nm). Recordings were performed in the presence of the generic glutamate receptor blocker kynurenic acid (3 mm) to isolate slow responses. Inset, Example of a firing pattern of a BA IN exhibiting the light-evoked slow EPSC. ***B***, Slow EPSC evoked in BA INs is blocked by the 5-HT_2A_ receptor antagonist MDL 100907 (1 μm; paired *t* test, *p* = 0.0018, *n* = 9). ***C***, Slow EPSC amplitude is increased significantly by higher stimulation frequencies. Slow EPSC amplitudes are normalized to the amplitude evoked at 20 Hz stimulation frequency for each cell (one-way ANOVA, *p* < 0.0001; Bonferroni's *post hoc* comparisons, 1 Hz vs 10 Hz *p* = 0.0368, 10 Hz vs 20 Hz *p* = 0.0139, 1 Hz vs 20 Hz *p* = 0.0020; *n* = 6). ***D***, Example of a voltage-clamp (−70 mV) trace from a BA IN showing a slow IPSC evoked by 30 s optical stimulation at 20 Hz of DRN axons. The slow IPSC is blocked by the 5-HT_1A_ receptor antagonist WAY 100635 (1 μm). Recordings were performed in the presence of the generic glutamate receptor blocker kynurenic acid (3 mm) to isolate slow responses. Inset, Example of a firing pattern of a BA IN exhibiting the light-evoked slow IPSC. ***E***, Slow IPSC evoked in BA INs is blocked by the 5-HT_1A_ receptor antagonist WAY 100635 (1 μm; paired *t* test, *p* = 0.0391, *n* = 7). ***F***, Slow IPSC amplitude is significantly increased by higher stimulation frequencies. Slow IPSC amplitudes are normalized to the amplitude evoked at 20 Hz stimulation frequency for each cell (one-way ANOVA, *p* = 0.0043; Bonferroni's *post hoc* comparisons, 1 Hz vs 20 Hz *p* = 0.0274; *n* = 5). ***G***, Representative voltage-clamp (−70 mV) traces from a BA IN showing simultaneous fast EPSCs and a slow IPSC evoked by optical stimulation at 20 Hz (left), at short (first 10 light pulses, top traces) and long (entire 20 Hz train, bottom traces) time scales. The fast EPSCs are blocked by AMPA/kainate and NMDA receptor antagonists NBQX (10 μm) and APV (50 μm; middle). Both the fast EPSCs and the slow IPSC are blocked by a combination of NBQX, APV, and WAY 100635 (right). ***H***, Light-evoked fast EPSCs and slow IPSC cooccurring in BA INs (left, *n* = 3) are blocked by glutamate receptor (*middle*, *n* = 2) and 5-HT_1A_ antagonists (right, *n* = 2), respectively. Data are expressed as means ± SEM. *p < 0.05; **p < 0.01.

Because single pulses of light did not evoke any detectable non-glutamate-mediated response in BA INs, further experiments tested the effect of light activation of 5-HT neurons under conditions in which glutamatergic transmission was blocked with kynurenic acid (3 mm). Notably, under these conditions, light application at a higher frequency (20 Hz) evoked both excitatory and inhibitory effects in the form of slow EPSCs and IPSCs, which were significantly different from baseline in 53% and 37% of INs tested, respectively (Fig. 3*A*,*D*). The slow EPSC outlasted the duration of the stimulation train and was blocked by the selective 5-HT_2A_ receptor antagonist MDL 100907 (150 nm; paired *t* test, *p* = 0.0018, *n* = 9; Fig. 3*B*). In comparison, the slow IPSC declined before the end of the stimulation train and was blocked by the selective 5-HT_1A_ receptor antagonist WAY 100635 (1 μm; paired *t* test, *p* = 0.0391, *n* = 7; Fig. 3*E*). Therefore, light-activated release of 5-HT can both excite and inhibit BA INs via 5-HT_2A_ and 5-HT_1A_ receptors, respectively.

Next, the sensitivity of these slow 5-HT-mediated responses to stimulation frequency was tested (1, 10, and 20 Hz). The peak amplitudes of both the slow EPSC and slow IPSC increased with higher frequencies of stimulation (one-way ANOVA; slow EPSC, *p* < 0.0001, *n* = 6; slow IPSC, *p* = 0.0043, *n* = 7; Fig. 3*C*,*F*), suggesting that, in contrast to glutamate signaling, the strength of 5-HT-mediated signaling onto BA INs is greatest at higher-frequency firing of 5-HT neurons.

In a subset of INs demonstrating both fast glutamatergic EPSCs and slow 5-HT_1A_-receptor-mediated IPSCs, 20 Hz optical stimulation was delivered without pharmacological blockade of glutamatergic transmission. This allowed examination of the relationship between glutamate and 5-HT release during the 20 Hz train. Although, at the beginning of the 20 Hz train, glutamatergic EPSCs were predominant, subsequent light pulses resulted in depression of the glutamatergic EPSCs and an increase in amplitude of the 5-HT_1A_-receptor-mediated IPSC, leading to a greater balance between glutamatergic and 5-HT transmission (Fig. 3*G*,*H*). These data suggest that the firing of DRN 5-HT neurons can shift the balance between glutamatergic and 5-HT modulation of BA INs dynamically.

### Heterogeneity of BA IN response to activation of 5-HT neurons

**Figure 4.**
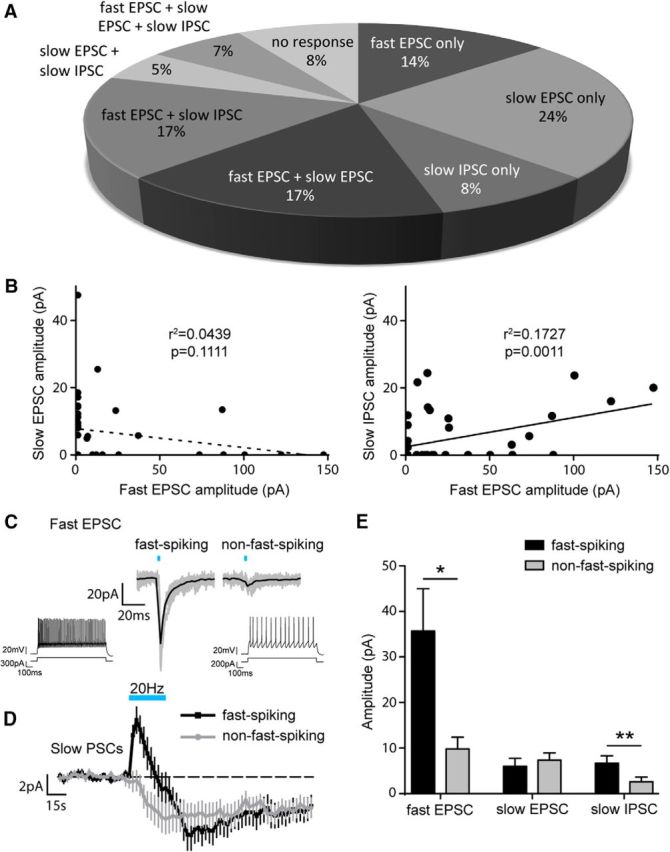
5-HT neurons differentially modulate BA INs. ***A***, Relative distribution (as a percentage) of response types in BA INs evoked by either single pulses (fast EPSC) or trains (slow EPSC and IPSC) of optical stimulation of DRN axons (*n* = 59). ***B***, No correlation between amplitudes of the fast EPSC and slow EPSC responses evoked in BA INs (Pearson correlation coefficient, *n* = 59; left). Positive correlation between amplitudes of the fast EPSC and slow IPSC responses evoked in BA INs (Pearson correlation coefficient, *n* = 59; right). ***C***, Example of voltage-clamp (−70 mV) traces showing a larger fast EPSC evoked in a fast-spiking (left) than in a non-fast-spiking (right) BA IN by a single light pulse (3 ms) activation of DRN axons. Inset, Example of firing patterns of a fast-spiking (left) and a non-fast-spiking (right) BA IN. ***D***, Mean voltage-clamp (−70 mV) traces from fast-spiking and non-fast-spiking BA INs showing similar amplitudes of the slow EPSC but different amplitudes of the slow IPSC evoked by 30 s optical stimulation at 20 Hz of DRN axons (fast-spiking *n* = 21, non-fast-spiking *n* = 36). ***E***, Amplitudes of the fast EPSC and the slow IPSC, but not the slow EPSC, are greater in fast-spiking than in non-fast-spiking BA INs (fast EPSC, Mann–Whitney test, *p* = 0.0114; slow EPSC, Mann–Whitney test, *p* = 0.4922, fast-spiking *n* = 21, non-fast-spiking *n* = 36; slow IPSC, Mann–Whitney test, *p* = 0.0090; fast-spiking *n* = 21, non-fast-spiking *n* = 36). Data are expressed as means ± SEM. *p < 0.05; **p < 0.01.

Analysis of the response of each cell to optical activation of 5-HT neurons revealed that individual BA INs (*n* = 59) displayed different combinations of glutamate-mediated fast EPSC and 5-HT-mediated slow EPSC and IPSC (Fig. 4*A*). Therefore, some INs exhibited a response mediated by either glutamate alone (14%) or 5-HT alone (37%), whereas other INs demonstrated a mixture of the two (41%). Overall, the majority (84%) of INs tested were excited by 5-HT neuron activation (glutamate alone, 5-HT alone, or both), whereas a minority (8%) were solely inhibited (5-HT alone). Interestingly, the amplitude of the slow IPSC positively correlated with the amplitude of the fast EPSC (Pearson correlation coefficient, *r*^2^ = 0.1727, *p* = 0.0011, *n* = 59 cells), whereas the slow EPSC and fast EPSC did not (Pearson correlation coefficient, *r*^2^ = 0.0439, *p* = 0.1111, *n* = 59 cells; Fig. 4*B*). This suggests that those INs strongly excited by glutamate (at low 5-HT neuron firing frequencies) are also strongly inhibited by 5-HT (at high 5-HT neuron firing frequencies).

Interestingly, separation of data obtained from fast-spiking and non-fast-spiking INs revealed evidence of cell-specific effects of 5-HT neuron activation. Namely, fast-spiking INs showed larger amplitude fast EPSCs than non-fast-spiking INs (Mann–Whitney test, *p* = 0.0114, fast-spiking *n* = 21, non-fast-spiking *n* = 36; Fig. 4*C*,*E*). In addition, fast-spiking INs showed larger amplitude slow IPSCs compared with non-fast-spiking INs (Mann–Whitney test, *p* = 0.0090, fast-spiking *n* = 21, non-fast-spiking *n* = 36), whereas the amplitude of slow EPSCs did not differ (Mann–Whitney test, *p* = 0.4922, fast-spiking *n* = 21, non-fast-spiking *n* = 36; Fig. 4*D*,*E*). These data demonstrate that 5-HT neurons are more likely to recruit fast-spiking INs via the release of glutamate at low firing rates. In contrast, high rates of 5-HT neurons' firing appear to trigger primarily excitation in non-fast-spiking INs and biphasic inhibition–excitation in fast-spiking INs via 5-HT.

### Activation of 5-HT neurons evokes 5-HT-mediated responses in BA PNs

**Figure 5.**
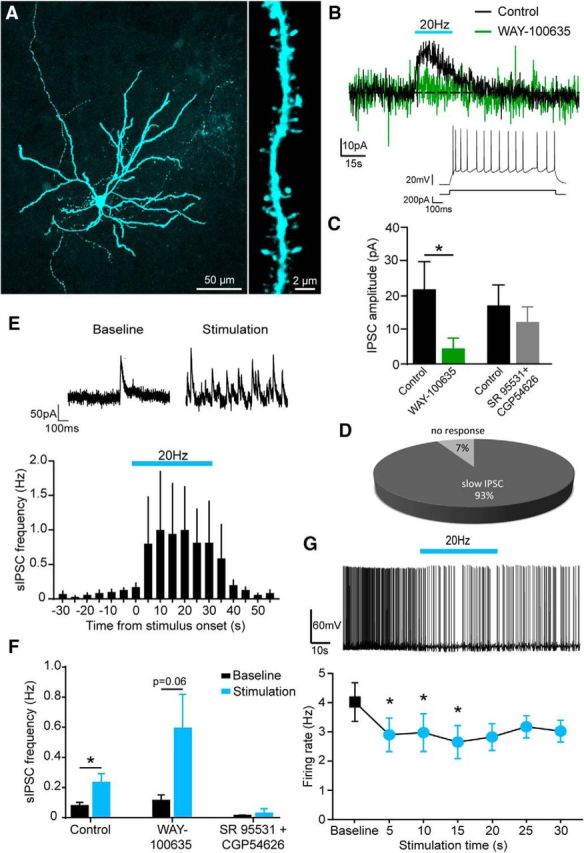
5-HT neurons inhibit BA PNs via 5-HT_1A_ receptors and enhanced GABA release. ***A***, Representative biocytin-filled PN showing a sparse axon with long-projecting branches (left) and spiny dendrites (right). ***B***, Example of a voltage-clamp (−50 mV) trace from a BA PN showing a slow IPSC evoked by 30 s optical stimulation at 20 Hz of DRN axons, blocked by the 5-HT_1A_ receptor antagonist WAY 100635 (1 μm). Inset, Stereotypical firing pattern of a BA PN exhibiting the light-evoked slow IPSC. ***C***, Slow IPSC evoked in BA PNs is blocked by the 5-HT_1A_ receptor antagonist WAY 100635 (1 μm; Wilcoxon matched-pairs signed-rank test, *p* = 0.0313, *n* = 7), but not by the GABA_A_ and GABA_B_ receptor antagonists SR 95531 (50 μm) and CGP54626 (5 μm; Wilcoxon matched-pairs signed-rank test, *p* = 0.6250, *n* = 5). ***D***, Relative distribution (as a percentage) of response types in BA PNs evoked by optical stimulation of 5-HT axons (*n* = 13). ***E***, Example of a voltage-clamp (−50 mV) trace from a BA PN showing an increase in sIPSC frequency during 20 Hz optical stimulation of DRN axons (top). Mean PSTH showing an increase in sIPSC frequency in BA PNs during 30 s optical stimulation at 20 Hz of DRN axons (*n* = 14; bottom) is shown. ***F***, Increase in sIPSC frequency in BA PNs is blocked by the GABA_A_ and GABA_B_ receptor antagonists SR 95531 (50 μm) and CGP54626 (5 μm), but not the 5-HT_1A_ antagonist WAY100635 (1 μm; control, Wilcoxon matched-pairs signed-rank test, *p* = 0.0254, *n* = 13; WAY 100635, paired *t* test, *p* = 0.0609, *n* = 7; SR 95531 and CGP54626, Wilcoxon matched-pairs signed-rank test, *p* > 0.9999, *n* = 5). ***G***, Top, Example of a current-clamp trace from a BA PN. Rheobase-positive current was constantly injected to evoke baseline firing. Thirty seconds of optical stimulation at 20 Hz of 5-HT axons reduced the firing rate. Bottom, Firing rate of BA PNs is significantly reduced by optical stimulation at 20 Hz of 5-HT axons (repeated-measures one-way ANOVA, main effect of stimulation time *p* = 0.0371; Dunnett's multiple-comparisons test, baseline vs 5 s *p* = 0.0261, baseline vs 10 s *p* = 0.0456, baseline vs 15 s *p* = 0.0410, *n* = 8). Data are expressed as means ± SEM. *p < 0.05.

A final set of experiments tested the influence of optical activation of 5-HT neurons on BA PNs (Fig. 5*A*), which form the main output of this nucleus and convey information to other brain structures. In contrast to INs, single pulses of light did not evoke fast EPSCs in PNs (*n* = 18), suggesting that glutamate-releasing 5-HT terminals do not target this cell type. However, as with some INs, a higher stimulation frequency (20 Hz) evoked a slow IPSC in BA PNs (Fig. 5*B*). This response to optical activation of 5-HT neurons was 5-HT-mediated because it was reduced by the selective 5-HT_1A_ receptor antagonist WAY 100635 (1 μm; Wilcoxon matched-pairs signed-rank test, *p* = 0.0313, *n* = 7; Fig. 5*B*,*C*), whereas combined GABA_A_ and GABA_B_ receptor blockade using SR 95531 (50 μm) and CGP54626 (5 μm) had no statistically significant effect (Fig. 5*C*). In contrast to INs, PNs demonstrated a uniform response to activation of 5-HT terminals, with 93% of PNs tested exhibiting a slow IPSC and the remainder displaying no response (Fig. 5*D*).

Given that high-frequency stimulation of 5-HT terminals elicited 5-HT-mediated EPSCs in BA INs, we predicted an increase in sIPSC frequency in PNs (Rainnie, 1999; Jiang et al., 2009; Bocchio et al., 2015). Indeed, optical activation at 20 Hz increased sIPSC frequency in PNs (baseline vs stimulation, Wilcoxon matched-pairs signed-rank test, *p* = 0.0254, *n* = 13; Fig. 5*E*). This effect was blocked by SR 95531 (50 μm) and CGP54626 (5 μm; baseline vs stimulation, Wilcoxon matched-pairs signed-rank test, *p* > 0.9999, *n* = 5), thereby demonstrating the GABAergic and disynaptic origin of these events. This was confirmed by the action of the 5-HT_1A_ receptor antagonist WAY 100635 (1 μm), which, as described above, abolished direct slow outward currents in INs. Consistent with this result, WAY 100635 tended to enhance, although not significantly, sIPSC frequency during stimulation compared with baseline (baseline vs stimulation, paired *t* test, *p* = 0.0609, *n* = 7; Fig. 5*F*). Increased sIPSC frequency therefore likely arose from 5-HT modulation of BA INs.

Finally, the impact of activation of 5-HT neurons on the firing of PNs was tested. To elicit firing in baseline conditions, positive rheobase current was injected into PNs in current-clamp mode. Under these conditions, optical stimulation at 20 Hz caused a significant reduction in PN firing rate (repeated measures one-way ANOVA, main effect of stimulation time *p* = 0.0371; Dunnett's multiple-comparisons test, baseline vs 5 s *p* = 0.0261, baseline vs 10 s *p* = 0.0456, baseline vs 15 s *p* = 0.0410, *n* = 8; Fig. 5*G*). Collectively, these observations suggest that the 5-HT input to the BA (at high firing rates) causes a direct 5-HT-mediated inhibition of PNs, as well as an indirect inhibition through increased GABAergic inputs from INs, leading to an overall decrease in output from the BA.

## Discussion

The current study investigated the action of physiologically released 5-HT on amygdala circuitry (specifically the BA) using a Cre-dependent construct that allowed selective optogenetic (ChR2) targeting of 5-HT neurons. Optical activation of 5-HT neurons elicited marked responses in BA neurons, which pharmacological analysis revealed to be mediated by a combination of 5-HT and ionotropic glutamate receptor-induced effects, suggesting 5-HT and glutamate co-release. Interestingly, this dual-transmitter signaling was separable in terms of stimulus frequency, with glutamate release occurring even at low frequencies, but 5-HT requiring higher frequencies. Furthermore, BA INs and PNs responded differently to 5-HT neuron activation, suggesting that 5-HT and glutamate signals targeted distinct BA cell types. Overall, the effect of activation of the 5-HT input to the BA was PN inhibition and thereby dampened BA output.

### Optogenetic control of 5-HT release

Expression of ChR2 in 5-HT neurons was achieved by delivering a viral vector carrying floxed ChR2 into the DRN of SERT-Cre mice (Zhuang et al., 2005). DRN ChR2-expressing cells were readily depolarized by pulses of light and were all 5-HT immunoreactive, verifying the high efficiency and selectivity of this construct, as reported recently (Cohen et al., 2015; Li et al., 2016).

Consistent with targeted expression of ChR2 to 5-HT neurons, optical stimulation evoked 5-HT-receptor-mediated responses in BA neurons. Specifically, a proportion of BA INs (53%) displayed a slow EPSC blocked by the 5-HT_2A_ receptor antagonist MDL 100907, whereas some INs (37%) responded with a slow IPSC blocked by the 5-HT_1A_ receptor antagonist WAY 100635. Conversely, optical stimulation evoked a 5-HT_1A_-receptor-mediated IPSC in almost all (93%) PNs tested, as well as an increase in indirect GABA-mediated inhibition of PNs, likely caused by depolarization of INs. Importantly, inhibition of PNs by 5-HT neurons was able to suppress PN firing.

These findings agree with previous pharmacological studies of the effect of bath-applied exogenous 5-HT on BA neurons. Bath-applied 5-HT evoked a 5-HT_2A_-receptor-mediated depolarization of the majority of INs that, in turn, led to increased sIPSC frequency in PNs (Rainnie, 1999; Jiang et al., 2009; Bocchio et al., 2015). In addition, exogenous 5-HT evoked hyperpolarization of a subset of PNs (Rainnie, 1999; Bocchio et al., 2015). The main difference between the effect of optically evoked and bath-applied 5-HT is the 5-HT_1A_-mediated inhibition of some INs and virtually all PNs observed here using the optogenetic method. Previous studies have emphasized the difference in time scale between pharmacological and optogenetic approaches and the potential for activation of nonsynaptic receptors by bath-applied transmitter (Unal et al., 2015). Here, the 5-HT_1A_-mediated responses showed desensitization within tens of seconds. Therefore, bath application could fail to detect these slow IPSCs, although bath-applied 5-HT_1A_-mediated effects have been reported in other amygdala regions (Bocchio et al., 2016a).

### Release of glutamate from 5-HT neurons

A key finding of our study is that optical activation evoked fast EPSCs in BA INs, an effect inhibited by NMDA/AMPA/kainate receptor antagonists. Although earlier immunohistochemical studies report the localization of ionotropic 5-HT_3A_ receptors on INs in the amygdala (Mascagni and McDonald, 2007), the optically evoked fast excitation was completely abolished by glutamate receptor blockade, thereby ruling out any involvement of 5-HT. The absence of 5-HT_3A_-receptor-mediated responses is consistent with the sparse expression of this receptor in BLA INs (Mascagni and McDonald, 2007).

To our knowledge, this is the first study demonstrating evidence of glutamate and 5-HT co-release in the BA. In a recent study combining optogenetics and patch-clamp recordings in the bed nucleus of the stria terminalis (SERT-Cre mice plus ChR2), evidence of glutamate release from 5-HT terminals was not observed (Marcinkiewcz et al., 2016). In contrast, another study reported that optical activation of 5-HT neurons (Pet1-Cre mice plus ChR2) evoked fast glutamate and slow 5-HT-receptor-mediated responses in the nucleus accumbens and ventral tegmental area (Liu et al., 2014). Moreover, a previous study reported evidence of 5-HT and glutamate co-release in the hippocampus, albeit using a ChR2-targeting approach that was not 5-HT selective (Varga et al., 2009). The latter two reports agree with earlier electrophysiological evidence that developing raphe 5-HT neurons in culture release both 5-HT and glutamate (Johnson, 1994).

This functional evidence of glutamate and 5-HT co-release is supported by molecular evidence for colocalization of VGLUT3 and 5-HT in DRN neurons in mice (Fu et al., 2010), as well as rats (Gras et al., 2002; Hioki et al., 2004) and humans (Vigneault et al., 2015). Furthermore, the current observation of VGLUT3 localization in 5-HT (ChR2/YFP+) axons in the BA agrees with similar findings in other regions including cortex, hippocampus, and striatum (Shutoh et al., 2008; Gagnon and Parent, 2014; Voisin et al., 2016).

Interestingly, we observed that some BA INs exhibited responses mediated by either glutamate alone (14%) or 5-HT alone (37%), whereas other INs responded to optical stimulation with a mixture of glutamate and 5-HT responses (41%). Because only 5-HT neurons were targeted optogenetically, this diversity of responses could arise from two mechanisms. First, this signaling could be coded postsynaptically: INs could possess different 5-HT and glutamate receptor expression profiles. In this case, 5-HT terminals would release 5-HT and glutamate from the same boutons, but whether the signal is 5-HT or glutamate mediated would be determined by the receptors expressed at the postsynaptic target. Alternatively, this signaling could be coded presynaptically: 5-HT axons could segregate glutamate- and 5-HT-containing vesicles within distinct boutons either completely or partially. This scenario is consistent with the relatively sparse expression of VGLUT3 in DRN 5-HT terminals. The ability to segregate or colocalize 5-HT- and glutamate-releasing vesicles would be an example of “cotransmission,” as suggested previously by Vaaga et al. (2014). Interestingly, cotransmission implies different calcium sensitivity for each neurotransmitter released (Ghijsen and Leenders, 2005), which is consistent with the present finding that glutamate- and 5-HT-mediated responses in the BA had differential sensitivity to stimulation frequency (see below).

### Frequency-dependent modulation of BA INs by 5-HT

As noted, the response of BA INs to glutamate had a different sensitivity to stimulation frequency compared with 5-HT responses. Specifically, glutamate-mediated fast excitations were observed at low stimulation frequencies (≤1 Hz) and response amplitude diminished with increased stimulation frequency (10–20 Hz), potentially due to exhaustion of vesicular stores or glutamate receptor desensitization. In contrast, 5-HT-mediated slow excitatory and inhibitory responses only emerged at the higher frequencies.

By examining glutamatergic EPSCs and 5-HT_1A_-receptor-mediated IPSCs concurrently during the 20 Hz stimulation trains, we confirmed that the glutamatergic response was strong at the beginning of the train, but depressed over time. Conversely, 5-HT responses were absent when initial light pulses were delivered, but increased over time. This suggests that both the firing rate and the duration of firing of 5-HT neurons are critical parameters controlling the balance between glutamate and 5-HT transmission in BA INs.

Because many DRN 5-HT neurons fire in a manner that decreases with sleep and increases with arousal (Veasey et al., 1995; Sakai, 2011), 5-HT neurons may signal principally via glutamate during some behavioral states (e.g., sleep), at least in the amygdala. It is important to keep in mind that DRN 5-HT neurons generally fire at slow rates (0.5–5 Hz; Allers and Sharp, 2003; Schweimer and Ungless, 2010; Sakai, 2011). Therefore, prolonged 20 Hz optical stimulation may result in supraphysiological 5-HT accumulation and 5-HT receptor activation. However, we observed significant 5-HT responses in BA neurons also at 10 Hz stimulation. Furthermore, recent opto-tagging studies in awake mice suggest that the firing of DRN 5-HT neurons increases markedly (15–20 Hz, with individual 5-HT neurons firing at up to 60 Hz) in response to punishing or rewarding stimuli (Cohen et al., 2015; Li et al., 2016). The current data argue that 5-HT neurons signal such behaviors in the amygdala, and possibly other regions, via prominent 5-HT release.

Interestingly, fast- and non-fast-spiking BA INs demonstrated a differential response to optical stimulation of 5-HT neurons, suggesting cell-specific targeting; fast-spiking INs displayed greater fast glutamatergic excitation and 5-HT_1A_-receptor-mediated inhibition than non-fast-spiking INs. Therefore, slower firing rates (0.5–5 Hz) of 5-HT neurons, for example, during wakefulness, could favor glutamatergic recruitment of fast-spiking BA INs. In contrast, higher firing rates (15–60 Hz), for example, during punishment/reward, may lead to more complex glutamate/5-HT modulation of fast- and non-fast-spiking INs. Future studies should determine how molecularly defined BA INs respond to low/high-frequency stimulation of 5-HT neurons. Importantly, the prominent 5-HT_1A_-receptor-mediated inhibition of fast-spiking INs is in agreement with an immunohistochemical study showing expression of 5-HT_1A_ receptors on parvalbumin-expressing INs (Aznar et al., 2003), some of which are fast spiking (Rainnie et al., 2006).

The present study does not establish whether similar rules of glutamate/5-HT cotransmission occur in the LA, which receives a sparse 5-HT innervation compared with the BA. Interestingly, VGLUT3 immunoreactivity is abundant in the BA, but not in the LA (Nickerson Poulin et al., 2006), suggesting that glutamate release by DRN 5-HT axons could be less prominent in this area, as also appears to be the case in the bed nucleus of the stria terminalis (Marcinkiewcz et al., 2016).

### Functional implications

The vast majority of BA PNs (93%) were inhibited directly by 5-HT release, suggesting that the main effect of 5-HT transmission in the BA is the suppression of its glutamatergic output. The inhibitory action of 5-HT neurons on PN firing could have a profound impact on emotional behavior because optogenetic inhibition of BLA PNs influences anxiety, fear learning, and appetitive learning through different projection targets (Tye et al., 2011; Senn et al., 2014; Namburi et al., 2015). However, the direct inhibition of PN firing might not be long lasting, because the 5-HT_1A_-receptor-mediated IPSCs decayed before the end of the optical stimulation, likely due to receptor desensitization. This suggests that long-lasting modulation of BA PNs by DRN 5-HT neurons can only occur via 5-HT_2A_-receptor-mediated (and potentially glutamate-mediated) depolarization of INs, which leads to enhanced GABA release onto PNs.

Overall, the present study suggests that DRN 5-HT neurons are able to fine-tune populations of BA INs across multiple timescales using firing rate, firing duration, and the different kinetics of glutamate and 5-HT_1A_ and 5-HT_2A_ receptors. This could result in redistribution of GABA release across different subcellular domains of BA PNs, a phenomenon that has been shown to influence emotional learning (Wolff et al., 2014).
